# Fur Seal Feces-Associated Circular DNA Virus Identified in Pigs in Anhui, China

**DOI:** 10.1007/s12250-020-00232-3

**Published:** 2020-06-02

**Authors:** Zhibin Shi, Chunguo Liu, Huanliang Yang, Yan Chen, Hua Liu, Lili Wei, Zaisi Liu, Yongping Jiang, Xijun He, Jingfei Wang

**Affiliations:** 1 State Key Laboratory of Veterinary Biotechnology and State Data Center for Animal Infectious Diseases, Harbin Veterinary Research Institute, Chinese Academy of Agricultural Sciences, Harbin, 150000 China; 2 Anhui Animal Diseases Prevention and Control Center and Key Laboratory of Veterinary Pathobiology and Disease Prevention and Control of Anhui Province, Hefei, 230000 China

**Keywords:** Fur seal feces-associated circular DNA virus (FSfaCV), Viral metagenomics, Pig, Genomic characterization, China

## Abstract

Fur seal feces-associated circular DNA virus (FSfaCV) is an unclassified circular replication-associated protein (Rep)-encoding single-stranded (CRESS) DNA virus that has been detected in mammals (fur seals and pigs). The biology and epidemiology of the virus remain largely unknown. To investigate the virus diversity among pigs in Anhui Province, China, we pooled 600 nasal samples in 2017 and detected viruses using viral metagenomic methods. From the assembled contigs, 12 showed notably high nucleotide acid sequence similarities to the genome sequences of FSfaCVs. Based on these sequences, a full-length genome sequence of the virus was then obtained using overlapping PCR and sequencing, and the virus was designated as FSfaCV-CHN (GenBank No. MK462122). This virus shared 91.3% and 90.9% genome-wide nucleotide sequence similarities with the New Zealand fur seal strain FSfaCV-as50 and the Japanese pig strain FSfaCV-JPN1, respectively. It also clustered with the two previously identified FSfaCVs in a unique branch in the phylogenetic tree based on the open reading frame 2 (ORF2), Rep-coding gene, and the genome of the reference CRESS DNA viruses. Further epidemiological investigation using samples collected in 2018 showed that the overall positive rate for the virus was 56.4% (111/197) in Anhui Province. This is the first report of FSfaCVs identified in pigs in China, and further epidemiological studies are warranted to evaluate the influence of the virus on pigs.

**Electronic supplementary material:**

The online version of this article (10.1007/s12250-020-00232-3) contains supplementary material, which is available to authorized users.

## Introduction

Many viruses with circular DNA genomes, including circular replication-associated protein (Rep)-encoding single-stranded (CRESS) DNA viruses, have been reported in both human and animal virome studies (Sikorski *et al.* 2013a; Tan le *et al.* 2013; Cui *et al.* 2017; Kaszab *et al.* 2018; Kraberger *et al.* 2018; Varsani and Krupovic 2018). These viruses are typically characterized by a small circular genome (< 10 kb) coated by capsid proteins, without an envelope structure. Eukaryote-infecting CRESS DNA viruses, such as porcine circovirus 2 (PCV2), have mainly been identified within the family *Circoviridae* (Gillespie *et al.* 2009; Meng 2013). However, a convincing causal relationship between the animal-associated viruses from other CRESS DNA viral families (e.g., *Smacoviridae* and *Genomoviridae*) and any disease, has yet to be documented (de Rezende *et al.* 2018; Steel *et al.* 2016; Walters *et al.* 2017). However, infections with these viruses may play a role in disorders of the host immune system and become an indirect factor for diseases caused by other pathogens (Shulman and Davidson 2017).

According to a recent report issued by the International Committee on Taxonomy of Viruses (ICTV) (https://talk.ictvonline.org/), eukaryote-infecting CRESS DNA viruses have been classified into six families: *Bacilladnaviridae*, *Geminiviridae*, *Nanoviridae*, *Genomoviridae*, *Circoviridae*, and *Smacoviridae*. The viruses of the families *Bacilladnaviridae*, *Geminiviridae,* and *Nanoviridae* have been demonstrated to infect diatoms and plants in previous studies (Tomaru *et al.* 2013; Heydarnejad *et al.* 2017; Zerbini *et al.* 2017), while members of the *Genomoviridae*, *Circoviridae*, and *Smacoviridae* families have been identified in the feces of a variety of vertebrates, including humans and pigs (Li *et al.* 2010; Krupovic *et al.* 2016; Rosario *et al.* 2017; Varsani and Krupovic 2018). There are obvious differences in the aspects of genome characteristics, evolution, and pathogenicity among CRESS DNA viruses (Malathi and Renuka Devi 2019).

In recent years, many unclassified CRESS DNA viruses have been discovered using viral metagenomic methods (Shan *et al.* 2011; Guo *et al.* 2018; Rosario *et al.* 2019; Wang *et al.* 2019). Fur seal feces associate circular DNA virus (FSfaCV) was first identified in the fecal samples of New Zealand fur seals in 2013 (Sikorski *et al.* 2013b). The second report of the virus was published in 2017, which reported that FSfaCVs were detected in stool specimens of pigs in Japan (Oba *et al.* 2017). The FSfaCV genome has two major open reading frames (ORFs) in opposite directions. ORF1 encodes a major structural capsid protein, while ORF2 encodes replication-associated proteins (Sikorski *et al.* 2013b). No further information about this virus is available.

In this study, we report for the first time the identification of FSfaCVs in pigs in Anhui Province, China. We also preliminarily described the genome characteristics and prevalence of these viruses in this region.

## Materials and Methods

### Sample Collection, Preparation, and Sequencing

**Fig. 1:**
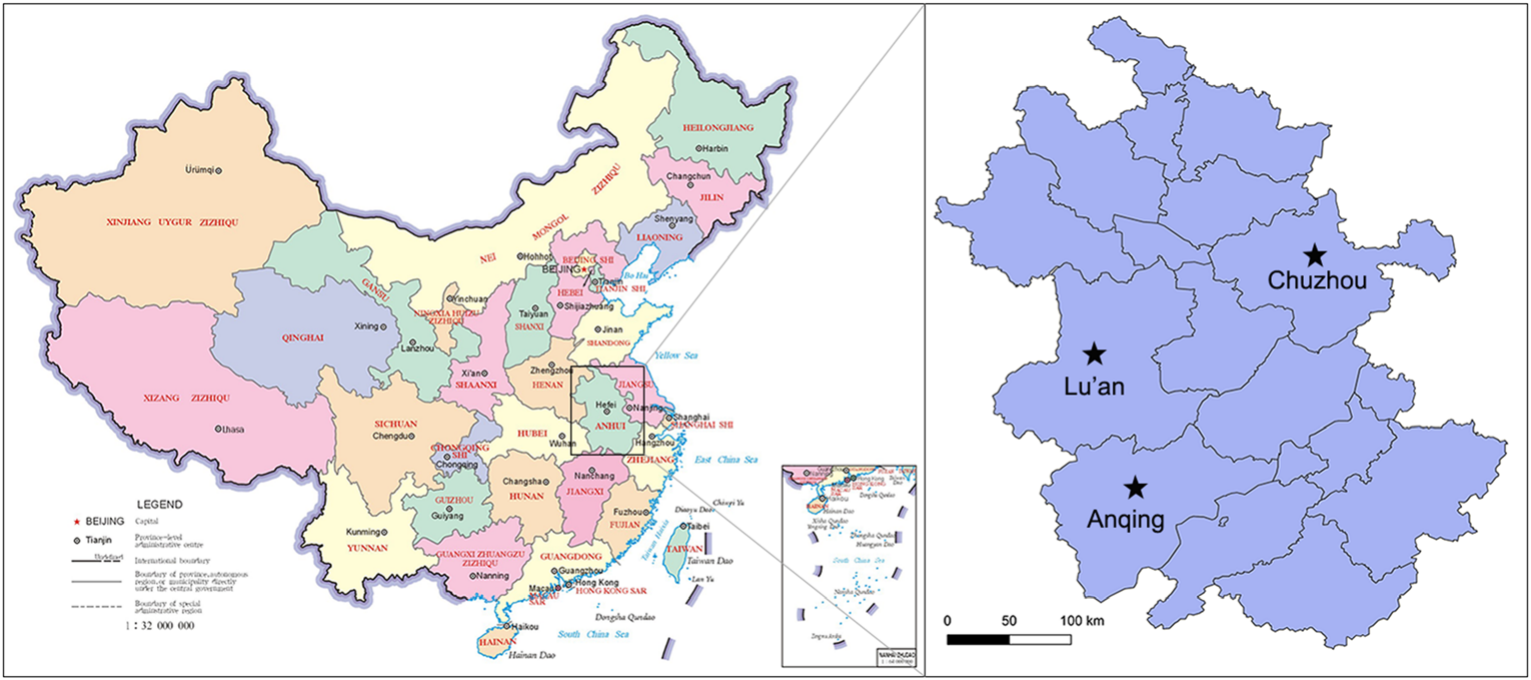
Sampling sites located in Anhui Province of China (Lu’an, Anqing, and Chuzhou city). The map was generated using QGIS Desktop 3.4.3 software.

A total of 600 nasal swabs were collected from a slaughterhouse during a surveillance of swine influenza in Anhui Province in 2017 (Fig. 1). Each of the nasal swabs was immersed in 1.5 mL sterile phosphate buffer saline (PBS), and half of the solution was used to detect the swine influenza virus. The remaining approximately 0.8 mL was pooled into one sample for virome analysis. Specimen processing and viral nucleic acid library preparation were performed as previously described (Shan *et al.* 2011). Briefly, the mixed samples were centrifuged at 13,000 × g for 20 min to remove impurities and filtered through 0.45-μm- and 0.22-μm-filter membranes. The pellet was dipped in PBS overnight after ultracentrifugation at 160,000 ×*g* for 4 h at 4 °C (Optima XPN-100 Ultracentrifuge, Beckman Coulter, Krefeld, Germany). The precipitates were repeatedly blended and dissolved in PBS. To remove the exogenous nucleic acid contamination, the sample was treated with DNase I and RNase I. Viral DNA/RNA was extracted from samples using EasyPure Viral DNA/RNA kit (TransGen, Beijing, China). Random PCR program was performed as follows. The first strand cDNA was synthesized with random primer of K9N: 5′-GACCATCTAGCGACCTCCCANNNNNNNNN-3′ and PrimeScript II RTase (Takara, Dalian, China) at 42 °C for 3 h, and then inactivated at 95 °C for 5 min. The second strand cDNA was synthesized with DNA Polymersae I Large (Klenow) Fragment (Promega, Madison, Wisconsin, USA) at 37 °C for 3 h, and then inactivated at 75 °C for 10 min. The DNA/cDNA was then amplified in a total reaction volume of 50 μL, which included 2 × KOD FX Neo buffer, 0.5 mmol/L each dNTP, 5 μL nucleotide, 10 mmol/L random primer of K9 (GACCATCTAGCGACCTCCCA) and 1 U KOD FX Neo DNA polymerase (Toyobo, Osaka, Japan). Finally, amplification was performed with 1 cycle of 94 °C for 2 min, followed by 40 cycles of 10 s at 98 °C, 30 s at 55 °C and 2 min at 68 °C. The PCR products were assessed by agarose gel electrophoresis. A total weight of 6 μg of random PCR products was submitted to Shanghai Personalbio Company and sequenced by Illumina HiSeq.

### Viral Metagenomic Analysis

Raw read data generated by Illumina sequencing were analyzed on a local viral metagenomic analysis platform. Briefly, the resulting reads were analyzed using the FastQC program (Brown *et al.* 2017) and the Cutadapt (Martin 2011) software to obtain clean data and were then assembled into contigs using the MEGAHIT software (Li *et al.* 2016) with default settings. The assembled contigs were noted by BLASTn (Zhang *et al.* 2000) with a cut-off E-value of 10^−5^ against a complete genome sequence database of all known viruses. We counted 12 contigs that showed high nucleotide sequence similarities with FSfaCV (Table 1).

**Table 1:** Contigs noted to belong to the genome of FSfaCVs.

Contig no.	Length (nt)	Description	Accession no.	E-value
Contig 277591	2774	FSfaCV isolate as50	KF246569	0
Contig 430548	2406	FSfaCV isolate as50	KF246569	0
Contig 24164	1041	FSfaCV isolate as50	KF246569	0
Contig 113448	961	FSfaCV isolate as50	KF246569	1.21E−163
Contig 5645	546	FSfaCV isolate as50	KF246569	2.70E−88
Contig 15591	1105	FSfaCV isolate as50	KF246569	2.70E−61
Contig 41875	398	FSfaCV isolate as50	KF246569	7.41E−43
Contig 170989	996	FSfaCV isolate as50	KF246569	3.21E−45
Contig 355689	342	FSfaCV isolate as50	KF246569	1.26E−94
Contig 142897	466	FSfaCV isolate as50	KF246569	1.08E−76
Contig 263359	334	FSfaCV isolate as50	KF246569	1.93E−137
Contig 87283	404	FSfaCV isolate as50	KF246569	6.92E−103
Contig 277591	2774	FSfaCV isolate JPN1	LC133373	0
Contig 430548	2406	FSfaCV isolate JPN1	LC133373	1.10E−172
Contig 24164	1041	FSfaCV isolate JPN1	LC133373	0
Contig 113448	961	FSfaCV isolate JPN1	LC133373	5.68E−157
Contig 5645	546	FSfaCV isolate JPN1	LC133373	5.75E−95
Contig 15591	1105	FSfaCV isolate JPN1	LC133373	1.25E−59
Contig 142897	466	FSfaCV isolate JPN1	LC133373	3.68E−111
Contig 355689	342	FSfaCV isolate JPN1	LC133373	1.27E−89
Contig 263359	334	FSfaCV isolate JPN1	LC133373	9.30E−111

### Determination of the full-length genome of FSfaCV

Based on the multiple sequence alignment results, we found that a gap region (approximately 2600–2780 nt) existed in the longest contig (contig 277591) compared to the genome sequence of the FSfaCV isolate as50. Therefore, overlapping PCR was performed to obtain the full-length genome of the virus. Briefly, a pair of primers (FSfaCV-F: 5′-CCAGTATGTTTTCCGATTG-3′; FSfaCV-R: 5′-CGCTTGTCCCTTATGTCTT-3′) were designed to amplify a 585-bp-long segment (containing part of the *Rep* and 5′-end intergenic region) in the FSfaCV genome, which completely covered the missing sequence of the contigs. The reaction was carried out in a 50-μL mixture including 25 μL of 2 × Taq PCR StarMix (GenStar), 2 μL of each primer (10 μmol/L), 2 μL of DNA template, and 19 μL of sterile water. The optimal PCR amplification procedure consisted of pre-denaturation at 95 °C for 2 min, followed by 40 cycles of denaturation at 98 °C for 10 s, annealing at 55 °C for 30 s, and extension at 72 °C for 40 s, followed by a final extension at 72 °C for 10 min. The PCR products were subjected to agarose gel electrophoresis (1.2%) in TAE buffer. The full-length genome sequence obtained was designated as FSfaCV-CHN. The genome sequence was deposited in the GenBank database under the accession number MK462122.

### Survey of FSfaCV Prevalence in Anhui Province

To confirm the viral metagenomic analysis results, a retrospective survey of FSfaCV prevalence in pigs in the Anhui Province was conducted. A total of 197 samples, consisting of 79 serum samples, 88 nasal swab samples, and 30 tissue samples (mixture of lymph node and spleen) were collected from clinically healthy pigs in three cities (Lu’an, Anqing, and Chuzhou) (Fig. 1). The details of the samples are shown in Table 2. The viral DNA was extracted from the samples using EasyPure Viral DNA/RNA kit (TransGen, Beijing). Gene segments of FSfaCV were then detected using the above-mentioned overlapping PCR method. Briefly, the reaction was carried out in a 20-μL mixture including 10 μL of 2 × Taq PCR StarMix (GenStar), 1 μL of each primer (10 μmol/L), 1 μL of DNA template, and 6 μL of sterile water. The optimal PCR amplification procedure was performed with three-step cycles as mentioned above. The PCR products were subjected to agarose gel electrophoresis (1.2%) in TAE buffer. From the FSfaCV-positive samples, 11 gene segments were cloned into the pMD18-T vector. These included seven serum samples—collected from Lu’an and Anqing—two nasal swab samples—collected from Lu’an—and two tissue samples collected from Chuzhou. Each ligation mixture was transformed into *Escherichia coli* DH5α competent cells and positive clones, with appropriate inserts being screened by colony PCR and subsequently sequenced by Kumei Comate Bioscience Co., Ltd. All of the gene fragments generated in this study were deposited in the GenBank database under the accession numbers MK462123–MK462133.

**Table 2:** The FSfaCV prevalence in pigs in 2017 of Anhui Province.

City location	Source	Sample type	Prevalence (%, positive/total)
Lu’an	Slaughterhouse A	Serum	30.0 (15/50)
		Nasal swab	52.3 (46/88)
Anqing	Farm A	Serum	100.0 (20/20)
	Farm B	Serum	100.0 (9/9)
Chuzhou	Slaughterhouse B	Lymph node and spleen	81.3 (13/16)
	Slaughterhouse C	Lymph node and spleen	57.1 (8/14)
Total			56.4 (111/197)

### Genome Organization of FSfaCV-CHN

The genome organization of the FSfaCV-CHN was generated using SeqBuilder software in the DNASTAR package (DNASTAR, Madison, WI, USA). The stem-loop structures were detected using the DNA folding prediction software Mfold (Zuker 2003). Open reading frames (ORFs) in the full genome of FSfaCV were identified using ORFfinder (http://www.ncbi.nlm.nih.gov/orffinder).

### Phylogenetic Analysis

To learn the phylogeny of the FSfaCVs obtained in this study, a multiple sequence alignment was performed with the amino acid sequence of the Rep of the new virus and reference strains, including FSfaCV-as50 (Sikorski *et al.* 2013b), FSfaCV-JPN1 (Oba *et al.* 2017), and 30 representative strains of the families *Circoviridae*, *Smacoviridae*, *Genomoviridae*, *Bacilladnaviridae*, *Geminiviridae*, and *Nanoviridae*. Maximum-likelihood trees based on Rep were then constructed with the default setting of the method. To explore the gene variations of the Chinese FSfaCVs, a neighbor-joining tree based on the 585-bp-long gene segments from the 11 positive samples obtained in this study and that of the FSfaCV isolates CHN, as50 (Sikorski *et al.* 2013b) and JPN1 (Oba *et al.* 2017) were also constructed. The methods used for constructing the phylogenetic trees were implemented in MEGA 6.06 (Tamura *et al.* 2013). The tree topologies were evaluated using 1000 bootstrap analyses.

## Results

### Identification of FSfaCV-CHN

Based on the results of the viral metagenomic analysis, 742,196 reads and 307 contigs were identified as belonging to viral genomes. Of these contigs, 12 showed very high nucleotide sequence similarities with the genome sequences of FSfaCVs evaluated using BLASTn with a cut-off E-value of 10^−5^ (Table 1). We then obtained the full-length genome sequence of a new virus based on the longest contig by using an overlapping PCR method. This genome sequence is 2915 nt in length, similar to that of the FSfaCV isolates JPN1 (LC133373) (2916 nt) and as50 (KF246569) (2925 nt). The genome-wide pairwise identities between the new virus and FSfaCV-as50 or FSfaCV-JPN1 were 91.3% and 90.9%, respectively. The genomic sequence alignment results of the three FSfaCVs are shown in Supplementary Figure S1. The amino acid length of the Rep (352 aa) of the Chinese strain is consistent with the other two strains, but the length of the Cap (375 aa) of the Chinese strain is slightly shorter than that of the other two strains (397 aa). The number of bases in the stem-loop structure at the 5′ UTR of the Chinese strain is the same as in the other two FSfaCVs. This result indicates that this genome sequence belongs to a fur seal feces-associated circular DNA virus, which we designated as FSfaCV-CHN.

### Prevalence of FSfaCV in Anhui Province

Based on the viral metagenomic analysis results, we conducted a retrospective survey of FSfaCV prevalence in pigs in Anhui Province. A total of 197 different samples were collected in November 2018. Using the PCR method developed in this study, we detected a total of 111 samples that were positive for the gene segment, yielding an overall prevalence of 56.4% (111/197), which was lower than the previously reported percentage for the virus in piglets in Japan (Oba *et al.* 2017). The positive rates of FSfaCVs in different samples—such as serum, nasal swab, or tissue—were variable. It was striking that sera from two farms in Anqing city were all positive (100%) for the virus. The details of the samples and the detection results are shown in Table 2. These results indicate that FSfaCVs have been circulating with a notably high prevalence in the pig population in Anhui.

### Genomic Structure of FSfaCV-CHN

**Fig. 2:**
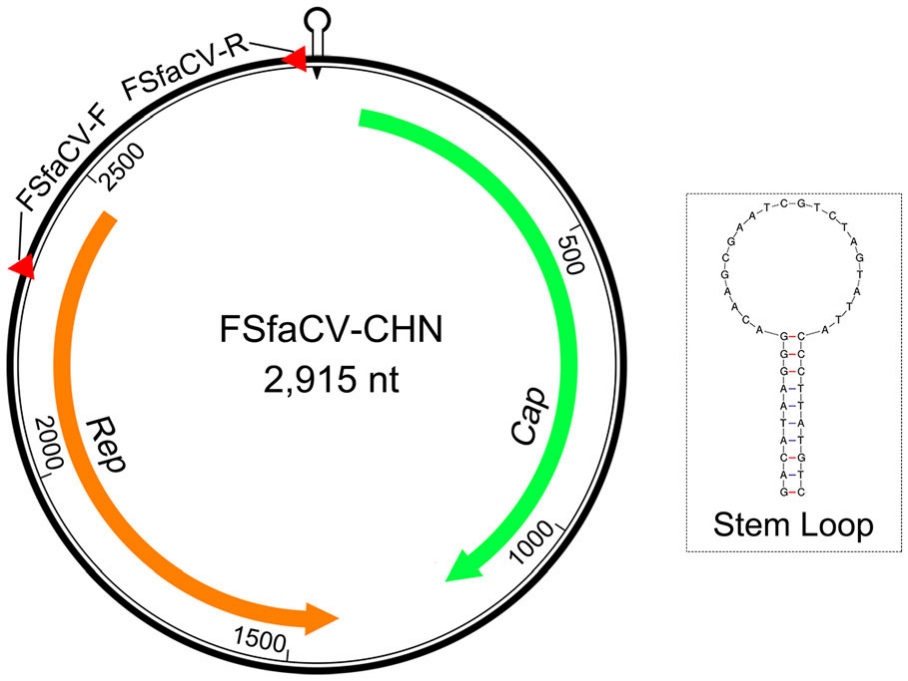
Genome organization of FSfaCV-CHN. *Cap* indicated by green arrow; *Rep* indicated by orange arrow. The positions and directions of the primers are marked by red triangles.

The FSfaCV-CHN genome contains two ORFs in opposite directions (Fig. 2). The ORF1 of FSfaCV-CHN is 1125 nt (81–1205) in length, encoding the capsid protein, while the replication-associated protein (Rep)-encoding gene ORF2 is 1056 nt (2470–1415) in length. Furthermore, the lengths of the intergenic regions between the 5′-ends of the *Rep* and *Cap* genes of the FSfaCVs are approximately 470 nt, and the 3′ intergenic regions between the stop codons of two major ORFs of the FSfaCVs are approximately 200 nt. A conserved nonanucleotide motif (5′-TAGTATTAC-3′) was also observed in the FSfaCVs and is located at the apex of the potential stem-loop structure. The *Rep* gene has been demonstrated to be able to introduce a breach to cleave the viral strand between positions 7 and 8 within the conserved nonanucleotide motif, probably initiating circovirus genome replication via rolling circle replication (RCR) (Rosario *et al.* 2017). These findings show that the genomic organization of FSfaCV-CHN is similar to that of the two previously identified FSfaCVs.

### Genetic Evolution of FSfaCVs

**Fig. 3:**
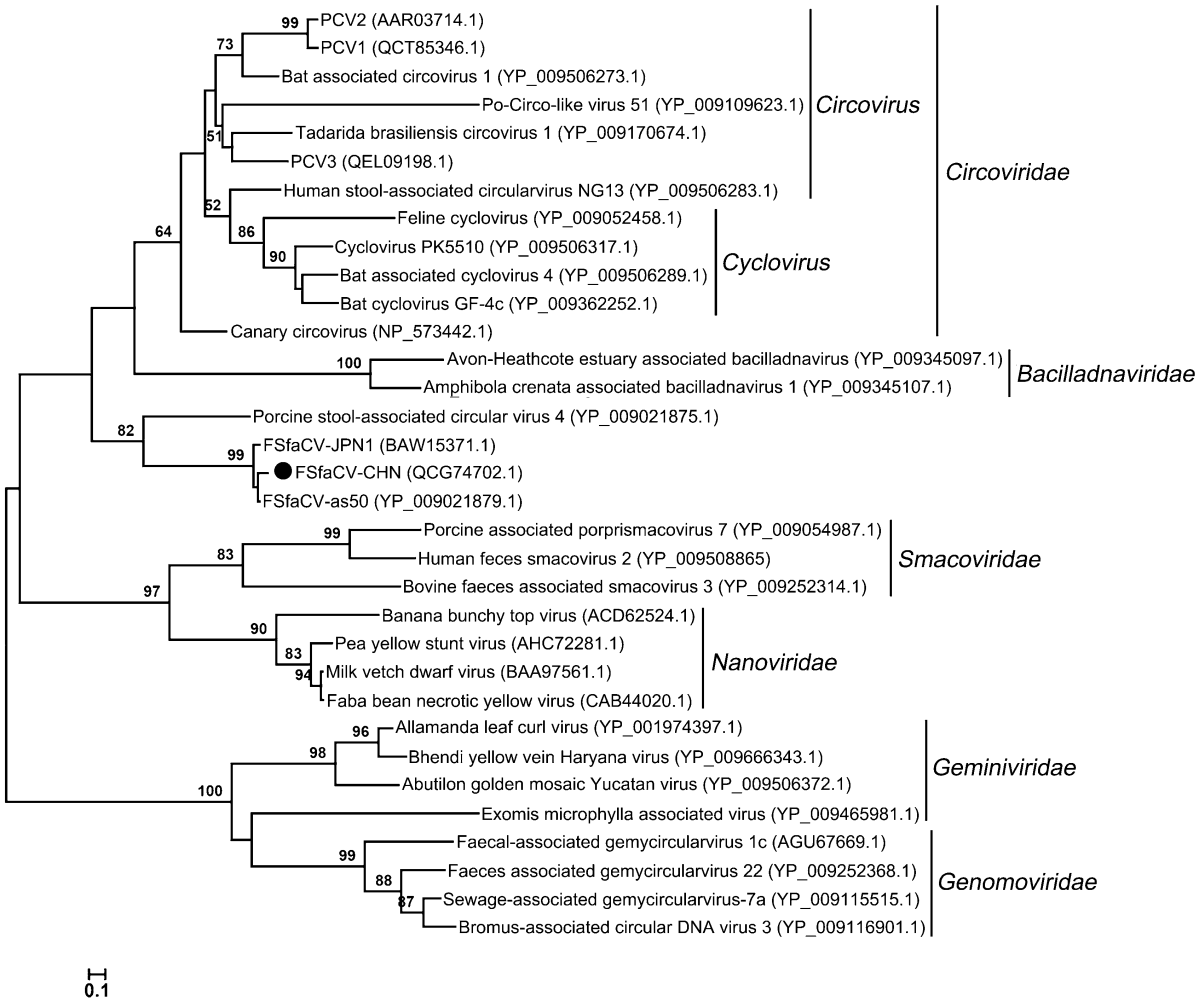
Maximum-Likelihood tree based on the Rep amino acid of CRESS DNA viruses. The tree was constructed using the Maximum-Likelihood method implemented in MEGA 6.06 with bootstrap tests of 1000 replicates. Viruses identified in this study are indicated with black dots. Sequences are labelled with virus names and amino acid accession numbers.

To further explore the phylogeny of the Chinese FSfaCV isolates, we constructed maximum likelihood trees based on the amino acid sequence of the Rep of FSfaCV-CHN and the reference strains. The three FSfaCVs were located in an independent clade in the Rep tree and were genetically closer to the families *Circoviridae* and *Bacilladnaviridae*, suggesting that they might have a common origin in phylogenetic evolution (Fig. 3). All of those FSfaCVs’ Rep shared < 55% amino acid identities with the members of other families (e.g., *Circoviridae*) (Rosario *et al.* 2017). These findings suggest that FSfaCVs may represent a novel family of CRESS DNA viruses.

**Fig. 4:**
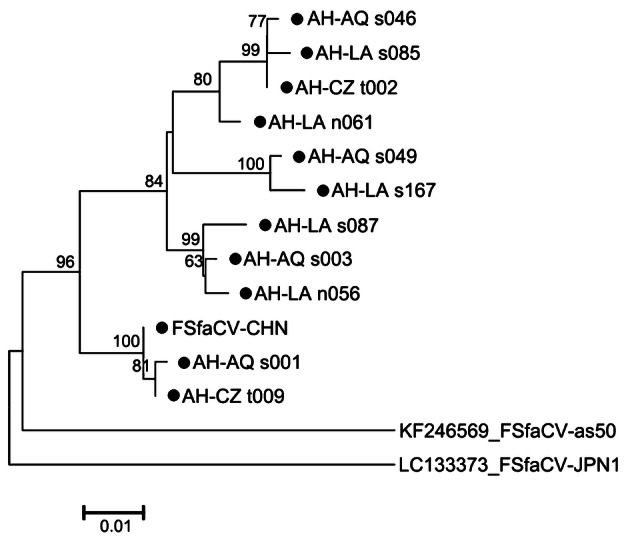
Phylogenetic tree based on 585-bp-long fragments (containing part of the *Rep* and 5′-end intergenic region) of the FSfaCVs. The tree was constructed using the neighbour-joining method implemented in MEGA 6.06 with bootstrap tests of 1000 replicates. Viruses identified in this study are indicated by black dots. All identified viruses except FSfaCV-CHN strain in this study come from the 111 positive samples. The virus name is composed of Province name (AH: Anhui), city name (AQ: Anqing; LA: Lu’an; CZ: Chuzhou), sample type (s: serum; n: nasal swab; t: tissue) and sample number.

To understand the potential gene variations among Chinese FSfaCVs, we sequenced gene fragments from 11 positive samples, and a neighbor-joining tree was built based on them and on sequences of the reference strains, including that of FSfaCV-CHN, FSfaCV-as50, and FSfaCV-JPN1. As indicated by the tree, all the Chinese viruses clustered into one branch but showed certain phylogenetic variations (Fig. 4). The pairwise nucleotide sequence identities of this gene fragment among all FSfaCVs were 88.7%–99.7%, which were 91.3%–99.7% among the Chinese strains. The results suggest that FSfaCVs in different geographical locations may have independent evolutionary traces.

## Discussion

CRESS DNA viruses are extremely diverse and widespread. They are “key players in the global virome” (Malathi and Renuka Devi 2019). However, their ecological or host impact remains largely unknown (Dayaram *et al.* 2015; Phan *et al.* 2016; Kraberger *et al.* 2018; Zhao *et al.* 2019). CRESS DNA viruses have been identified in various hosts, including bovines (Li *et al.* 2011), chimpanzees (Li *et al.* 2010), bats (Wu *et al.* 2016), chickens (Lima *et al.* 2017), humans (Phan *et al.* 2014; Phan *et al.* 2016; Cui *et al.* 2017), and pigs (Stenzel and Koncicki 2017; Zheng *et al.* 2017). As various new CRESS DNA viruses have been identified in pigs, our understanding of the diversity of these viruses has been enhanced. Some CRESS DNA viruses are important pathogens in pigs, such as porcine circovirus 2 (PCV2), which has been shown to be a potential pathogen of several syndromes known as porcine circovirus-associated disease, causing multisystemic infections in weaned and fattening pigs (Jiang *et al.* 2017). Therefore, the identification and characterization of novel CRESS DNA viruses is of great importance for evaluating the potential impacts of viruses on the pig industry.

The newly identified FSfaCVs in China showed similar genetic characteristics to the previously identified FSfaCVs. Compared to previous findings, the intergenic regions and the stem-loop structures showed significant differences between the FSfaCVs and other associated circular single-stranded DNA viruses (Krupovic *et al.* 2016; Rosario *et al.* 2017; Varsani and Krupovic 2018). These differences might be important evidence for distinguishing FSfaCVs from other associated-circular single-stranded DNA viruses. Furthermore, *Rep* gene is considered the only phylogenetic marker for CRESS DNA viruses and is widely used for phylogenetic analysis and high-level classification (Simmonds *et al.* 2017; Zhao *et al.* 2019). Based on the results of a phylogenetic analysis in which FSfaCVs were clustered together with a stool-associated circular virus within an independent clade on the phylogenetic tree and the identities of the amino acid sequence of the *Rep* gene between these FSfaCVs and the reference CRESS DNA viruses were all less than 55%. These findings suggest that a new family may be proposed to include FSfaCVs and other similar viruses.

FSfaCVs may infect multiple organs of pigs. In addition to previous studies that identified FSfaCVs from the feces of fur seals (Sikorski *et al.* 2013b) and from the feces and blood of healthy pigs (Oba *et al.* 2017), we detected FSfaCVs from nasal swabs, sera, and tissue specimens in healthy pigs, indicating that these viruses were distributed in multiple organs of pigs and that the viruses could also be excreted through respiratory droplets. These findings expand the body of knowledge concerning the biology and epidemiology of the virus. Furthermore, given that the virus was identified from healthy pigs in this study, we assume that this virus may be a component of the common virome community of pigs and may not be a direct pathogen, similar to a previous study on human anelloviruses (Maggi and Bendinelli 2010). Other studies, however, have shown that viruses without direct pathogenicity may weaken host immune function and trigger the infection of other pathogenic agents (Shulman and Davidson 2017).

To date, FSfaCVs have been identified in New Zealand, Japan, and China—regions that are geographically isolated from one another. The viruses identified in these regions have also shown unique genetic characteristics, which suggests that FSfaCVs may be distributed broadly around the world. In this study, an overall positive rate of 56.4% for the virus was detected in pigs in Anhui, China, and a 100% positive rate was found in two farms, suggesting that FSfaCVs might have been circulating with notably high prevalence in the pig population in Anhui. The full-length genome sequences of these 111 positive samples were not obtained, probably because of either the low virus loading in these samples or the amplification strategies we adopted. We are continuing to optimize the reaction conditions to obtain the genome sequences from these positive samples and other samples we collected more recently. Furthermore, although all of the viruses to date have been identified from clinically healthy pigs, given that the novel biological characteristics of these viruses and their pathogenicity in animals remain largely unknown, further research is needed to address the potential risks that these viruses pose to pigs in this region and in other places in China.

In summary, we have described the first identification, full genomic characterization, and preliminary epidemiological investigation of FSfaCVs in pigs in China.

## Electronic supplementary material

Below is the link to the electronic supplementary material.

Figure S1 Genome sequences comparison between FSfaCV-CHN, FSfaCV-JPN1 and FSfaCV-as50. Full length genome sequences of three FSfaCVs were aligned using ClustalW implemented in MEGA6.06. The coding and non-coding region are labeled, intergenic region (gray), *Cap* (green), and *Rep* (orange). All the nucleotides are shown in different colors. (PDF 3043 kb)

## References

[CR1] Brown J, Pirrung M, McCue LA (2017). FQC dashboard: integrates FastQC results into a web-based, interactive, and extensible FASTQ quality control tool. Bioinformatics.

[CR2] Cui L, Wu B, Zhu X, Guo X, Ge Y, Zhao K, Qi X, Shi Z, Zhu F, Sun L, Zhou M (2017). Identification and genetic characterization of a novel circular single-stranded DNA virus in a human upper respiratory tract sample. Arch Virol.

[CR3] Dayaram A, Goldstien S, Arguello-Astorga GR, Zawar-Reza P, Gomez C, Harding JS, Varsani A (2015). Diverse small circular DNA viruses circulating amongst estuarine molluscs. Infect Genet Evol.

[CR4] de Rezende RR, Mar TB, Paez LMC, Silva Xavier AD, Xavier CAD, Navas-Castillo J, Zerbini FM, Alfenas-Zerbini P (2018). Complete genome sequences of two gemycircularviruses associated with non-cultivated plants in Brazil. Arch Virol.

[CR5] Gillespie J, Opriessnig T, Meng XJ, Pelzer K, Buechner-Maxwell V (2009). Porcine circovirus type 2 and porcine circovirus-associated disease. J Vet Intern Med.

[CR6] Guo Z, He Q, Tang C, Zhang B, Yue H (2018). Identification and genomic characterization of a novel CRESS DNA virus from a calf with severe hemorrhagic enteritis in China. Virus Res.

[CR7] Heydarnejad J, Kamali M, Massumi H, Kvarnheden A, Male MF, Kraberger S, Stainton D, Martin DP, Varsani A (2017). Identification of a nanovirus-alphasatellite complex in *Sophora alopecuroides*. Virus Res.

[CR8] Jiang CG, Wang G, Tu YB, Liu YG, Wang SJ, Cai XH, An TQ (2017). Genetic analysis of porcine circovirus type 2 in China. Arch Virol.

[CR9] Kaszab E, Marton S, Forro B, Bali K, Lengyel G, Banyai K, Feher E (2018). Characterization of the genomic sequence of a novel CRESS DNA virus identified in Eurasian jay (*Garrulus glandarius*). Arch Virol.

[CR10] Kraberger S, Waits K, Ivan J, Newkirk E, VandeWoude S, Varsani A (2018). Identification of circular single-stranded DNA viruses in faecal samples of Canada lynx (*Lynx canadensis*), moose (*Alces alces*) and snowshoe hare (*Lepus americanus*) inhabiting the Colorado San Juan Mountains. Infect Genet Evol.

[CR11] Krupovic M, Ghabrial SA, Jiang D, Varsani A (2016). Genomoviridae: a new family of widespread single-stranded DNA viruses. Arch Virol.

[CR12] Li L, Kapoor A, Slikas B, Bamidele OS, Wang C, Shaukat S, Masroor MA, Wilson ML, Ndjango JB, Peeters M, Gross-Camp ND, Muller MN, Hahn BH, Wolfe ND, Triki H, Bartkus J, Zaidi SZ, Delwart E (2010). Multiple diverse circoviruses infect farm animals and are commonly found in human and chimpanzee feces. J Virol.

[CR13] Li L, Shan T, Soji OB, Alam MM, Kunz TH, Zaidi SZ, Delwart E (2011). Possible cross-species transmission of circoviruses and cycloviruses among farm animals. J Gen Virol.

[CR14] Li D, Luo R, Liu CM, Leung CM, Ting HF, Sadakane K, Yamashita H, Lam TW (2016). MEGAHIT v1.0: a fast and scalable metagenome assembler driven by advanced methodologies and community practices. Methods.

[CR15] Lima DA, Cibulski SP, Finkler F, Teixeira TF, Varela APM, Cerva C, Loiko MR, Scheffer CM, Dos Santos HF, Mayer FQ, Roehe PM (2017). Faecal virome of healthy chickens reveals a large diversity of the eukaryote viral community, including novel circular ssDNA viruses. J Gen Virol.

[CR16] Maggi F, Bendinelli M (2010). Human anelloviruses and the central nervous system. Rev Med Virol.

[CR17] Malathi VG, Renuka Devi P (2019). ssDNA viruses: key players in global virome. Virusdisease.

[CR18] Martin M (2011). Cutadapt removes adapter sequences from high-throughput sequencing reads. EMBnet J.

[CR19] Meng XJ (2013). Porcine circovirus type 2 (PCV2): pathogenesis and interaction with the immune system. Annu Rev Anim Biosci.

[CR20] Oba M, Katayama Y, Naoi Y, Tsuchiaka S, Omatsu T, Okumura A, Nagai M, Mizutani T (2017). Discovery of fur seal feces-associated circular DNA virus in swine feces in Japan. J Vet Med Sci.

[CR21] Phan TG, Luchsinger V, Avendano LF, Deng X, Delwart E (2014). Cyclovirus in nasopharyngeal aspirates of Chilean children with respiratory infections. J Gen Virol.

[CR22] Phan TG, da Costa AC, Del Valle Mendoza J, Bucardo-Rivera F, Nordgren J, O’Ryan M, Deng X, Delwart E (2016). The fecal virome of South and Central American children with diarrhea includes small circular DNA viral genomes of unknown origin. Arch Virol.

[CR23] Rosario K, Breitbart M, Harrach B, Segales J, Delwart E, Biagini P, Varsani A (2017). Revisiting the taxonomy of the family Circoviridae: establishment of the genus Cyclovirus and removal of the genus Gyrovirus. Arch Virol.

[CR24] Rosario K, Morrison CM, Mettel KA, Betancourt WQ (2019). Novel circular rep-encoding single-stranded DNA viruses detected in treated wastewater. Microbiol Resour Announc.

[CR25] Shan T, Li L, Simmonds P, Wang C, Moeser A, Delwart E (2011). The fecal virome of pigs on a high-density farm. J Virol.

[CR26] Shulman LM, Davidson I (2017). Viruses with circular single-stranded DNA genomes are everywhere!. Annu Rev Virol.

[CR27] Sikorski A, Arguello-Astorga GR, Dayaram A, Dobson RC, Varsani A (2013). Discovery of a novel circular single-stranded DNA virus from porcine faeces. Arch Virol.

[CR28] Sikorski A, Dayaram A, Varsani A (2013). Identification of a novel circular DNA virus in New Zealand fur seal (*Arctocephalus forsteri*) fecal matter. Genome Announc.

[CR29] Simmonds P, Adams MJ, Benko M, Breitbart M, Brister JR, Carstens EB, Davison AJ, Delwart E, Gorbalenya AE, Harrach B, Hull R, King AM, Koonin EV, Krupovic M, Kuhn JH, Lefkowitz EJ, Nibert ML, Orton R, Roossinck MJ, Sabanadzovic S, Sullivan MB, Suttle CA, Tesh RB, van der Vlugt RA, Varsani A, Zerbini FM (2017). Consensus statement: virus taxonomy in the age of metagenomics. Nat Rev Microbiol.

[CR30] Steel O, Kraberger S, Sikorski A, Young LM, Catchpole RJ, Stevens AJ, Ladley JJ, Coray DS, Stainton D, Dayaram A, Julian L, van Bysterveldt K, Varsani A (2016). Circular replication-associated protein encoding DNA viruses identified in the faecal matter of various animals in New Zealand. Infect Genet Evol.

[CR31] Stenzel T, Koncicki A (2017). The epidemiology, molecular characterization and clinical pathology of circovirus infections in pigeons—current knowledge. Vet Q.

[CR32] Tamura K, Stecher G, Peterson D, Filipski A, Kumar S (2013). MEGA6: molecular evolutionary genetics analysis version 6.0. Mol Biol Evol.

[CR33] Tan le V, van Doorn HR, Nghia HD, Chau TT, le Tu TP, de Vries M, Canuti M, Deijs M, Jebbink MF, Baker S, Bryant JE, Tham NT, BKrong Nguyen C, Boni MF, Loi TQ, le Phuong T, Verhoeven JT, Crusat M, Jeeninga RE, Schultsz C, Chau NV, Hien TT, van der Hoek L, Farrar J, de Jong MD (2013). Identification of a new cyclovirus in cerebrospinal fluid of patients with acute central nervous system infections. mBio.

[CR34] Tomaru Y, Toyoda K, Suzuki H, Nagumo T, Kimura K, Takao Y (2013). New single-stranded DNA virus with a unique genomic structure that infects marine diatom *Chaetoceros setoensis*. Sci Rep.

[CR35] Varsani A, Krupovic M (2018). Smacoviridae: a new family of animal-associated single-stranded DNA viruses. Arch Virol.

[CR36] Walters M, Bawuro M, Christopher A, Knight A, Kraberger S, Stainton D, Chapman H, Varsani A (2017). Novel single-stranded DNA Virus genomes recovered from chimpanzee feces sampled from the Mambilla Plateau in Nigeria. Genome Announc.

[CR37] Wang Y, Yang S, Liu D, Zhou C, Li W, Lin Y, Wang X, Shen Q, Wang H, Li C, Zong M, Ding Y, Song Q, Deng X, Qi D, Zhang W, Delwart E (2019). The fecal virome of red-crowned cranes. Arch Virol.

[CR38] Wu Z, Yang L, Ren X, He G, Zhang J, Yang J, Qian Z, Dong J, Sun L, Zhu Y, Du J, Yang F, Zhang S, Jin Q (2016). Deciphering the bat virome catalog to better understand the ecological diversity of bat viruses and the bat origin of emerging infectious diseases. ISME J.

[CR39] Zerbini FM, Briddon RW, Idris A, Martin DP, Moriones E, Navas-Castillo J, Rivera-Bustamante R, Roumagnac P, Varsani A, Ictv Report C (2017). ICTV virus taxonomy profile: geminiviridae. J Gen Virol.

[CR40] Zhang Z, Schwartz S, Wagner L, Miller W (2000). A greedy algorithm for aligning DNA sequences. J Comput Biol.

[CR41] Zhao L, Rosario K, Breitbart M, Duffy S (2019). Eukaryotic circular rep-encoding single-stranded DNA (CRESS DNA) viruses: ubiquitous viruses with small genomes and a diverse host range. Adv Virus Res.

[CR42] Zheng S, Wu X, Zhang L, Xin C, Liu Y, Shi J, Peng Z, Xu S, Fu F, Yu J, Sun W, Xu S, Li J, Wang J (2017). The occurrence of porcine circovirus 3 without clinical infection signs in Shandong Province. Transbound Emerg Dis.

[CR43] Zuker M (2003). Mfold web server for nucleic acid folding and hybridization prediction. Nucleic Acids Res.

