# Supplementary material for: Fur Seal Feces-Associated Circular DNA Virus Identified in Pigs in Anhui, China
Source: Virol Sin. 2020 Jun 2;36(1):25–32. doi: 10.1007/s12250-020-00232-3 (PMC7973343; doi:10.1007/s12250-020-00232-3)
Supplement: Supplementary file 1 — Genome sequences comparison between FSfaCV-CHN, FSfaCV-JPN1 and FSfaCV-as50. Full length genome sequences of three FSfaCVs were aligned using ClustalW implemented in MEGA6.06. The coding and non-coding region are labeled, intergenic region (gray), Cap (green), and Rep (orange). All the nucleotides are shown in different colors. (PDF 3043 kb) [file 12250_2020_232_MOESM1_ESM.pdf]

VIROLOGICA SINICA

Electronic Supplementary Material

Fur Seal Feces-Associated Circular DNA Virus Identified in Pigs in Anhui, China

Zhibin Shi[1] • Chunguo Liu[1] • Huanliang Yang[1] • Yan Chen[1] • Hua Liu[2] • Lili Wei[1] • Zaisi Liu[1] • Yongping Jiang[1] • Xijun He[1] • Jingfei Wang[1]✉

1. State Key Laboratory of Veterinary Biotechnology and State Data Center for Animal Infectious Diseases, Harbin Veterinary Research Institute, Chinese Academy of Agricultural Sciences, Harbin 150000, China.
2. Anhui Animal Diseases Prevention and Control Center and Key Laboratory of Veterinary Pathobiology and Disease Prevention and Control of Anhui Province, Hefei 230000, China.

Supporting information to DOI: 10.1007/s12250-020-00232-3

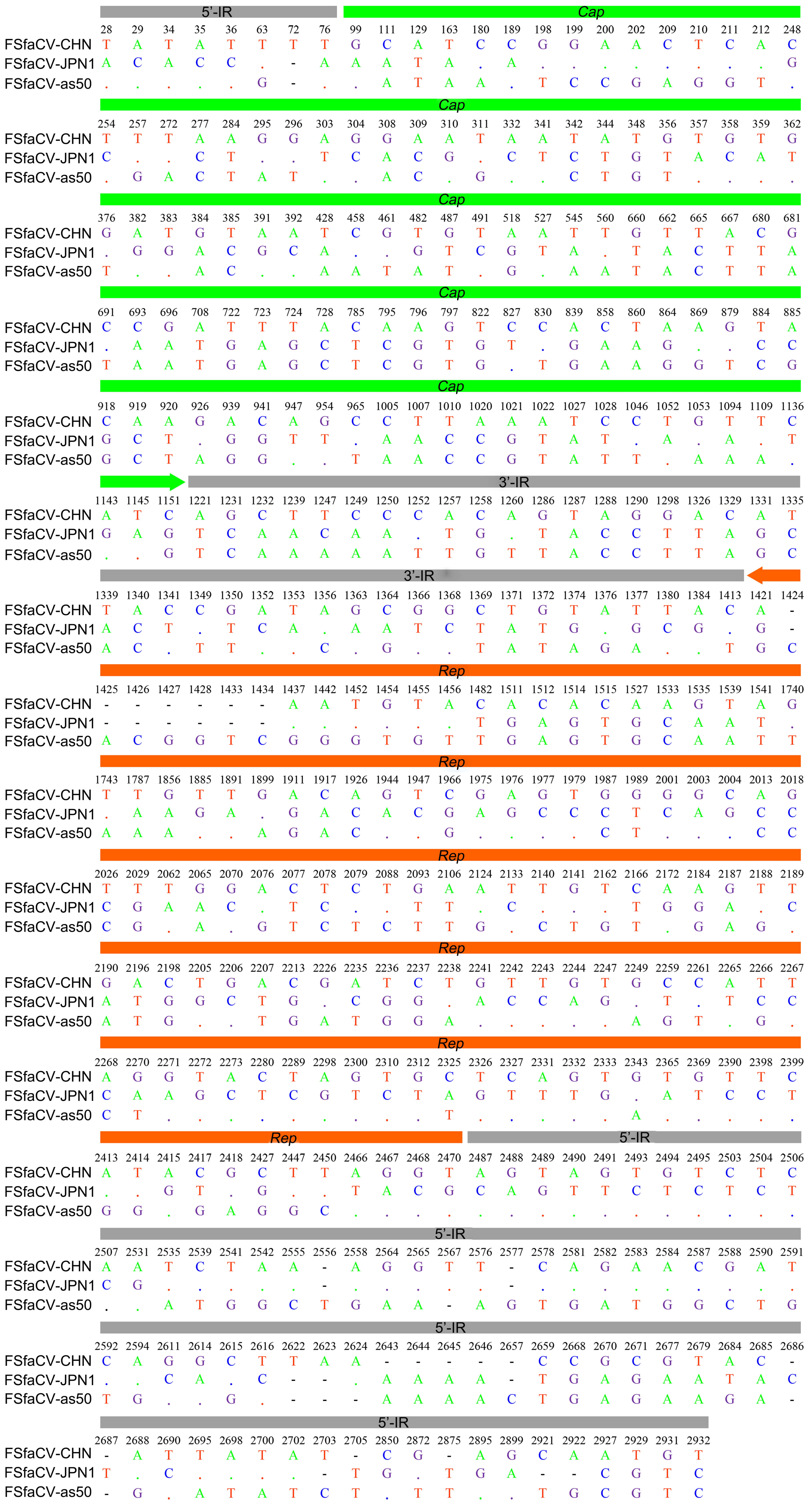
5'-IR
Cap
3'-IR
Rep
FSfaCV-CHN
FSfaCV-JPN1
FSfaCV-as50

Fig. S1. Genome sequences comparison between FSfaCV-CHN, FSfaCV-JPN1 and FSfaCV-as50. Full length genome sequences of three FSfaCVs were aligned using ClustalW implemented in MEGA6.06. The coding and non-coding region are labeled, intergenic region (gray), Cap (green), and Rep (orange). All the nucleotides are shown in different colors.
